# Comparative Transcriptional Analysis of Homologous Pathogenic and Non-Pathogenic *Lawsonia intracellularis* Isolates in Infected Porcine Cells

**DOI:** 10.1371/journal.pone.0046708

**Published:** 2012-10-03

**Authors:** Fabio A. Vannucci, Douglas N. Foster, Connie J. Gebhart

**Affiliations:** 1 Department of Veterinary and Biomedical Science, College of Veterinary Medicine, University of Minnesota, St. Paul, Minnesota, United States of America; 2 Department of Animal Science, College of Food, Agricultural and Natural Resource Science, University of Minnesota, St. Paul, Minnesota, United States of America; Cornell University, United States of America

## Abstract

*Lawsonia intracellularis* is the causative agent of proliferative enteropathy. This disease affects various animal species, including nonhuman primates, has been endemic in pigs, and is an emerging concern in horses. Non-pathogenic variants obtained through multiple passages in vitro do not induce disease, but bacterial isolates at low passage induce clinical and pathological changes. We hypothesize that genes differentially expressed between pathogenic (passage 10) and non-pathogenic (passage 60) *L. intracellularis* isolates encode potential bacterial virulence factors. The present study used high-throughput sequencing technology to characterize the transcriptional profiling of a pathogenic and a non-pathogenic homologous *L. intracellularis* variant during *in vitro* infection. A total of 401 genes were exclusively expressed by the pathogenic variant. Plasmid-encoded genes and those involved in membrane transporter (e.g. ATP-binding cassette), adaptation and stress response (e.g. transcriptional regulators) were the categories mostly responsible for this wider transcriptional landscape. The entire gene repertoire of plasmid A was repressed in the non-pathogenic variant suggesting its relevant role in the virulence phenotype of the pathogenic variant. Of the 319 genes which were commonly expressed in both pathogenic and non-pathogenic variants, no significant difference was observed by comparing their normalized transcription levels (fold change±2; p<0.05). Unexpectedly, these genes demonstrated a positive correlation (r^2^ = 0.81; p<0.05), indicating the involvement of gene silencing (switching off) mechanisms to attenuate virulence properties of the pathogenic variant during multiple cell passages. Following the validation of these results by reverse transcriptase-quantitative PCR using ten selected genes, the present study represents the first report characterizing the transcriptional profile of *L. intracellularis*. The complexity of the virulence phenotype was demonstrated by the diversity of genes exclusively expressed in the pathogenic isolate. The results support our hypothesis and provide the basis for prospective mechanistic studies regarding specific roles of target genes involved in the pathogenesis, diagnosis and control of proliferative enteropathy.

## Introduction

*Lawsonia intracellularis* is a fastidious intracellular bacterium and the etiologic agent of proliferative enteropathy (PE), an intestinal hyperplasic disease characterized by thickening of the mucosa of the intestine due to enterocyte proliferation [1]. Cell proliferation is directly associated with bacterial infection and replication in the intestinal epithelium [2]. As a result, mild to severe diarrhea is the major clinical sign described in infected animals [3]. Since the 1990s, PE has been endemic in swine herds and has been occasionally reported in various other species, including nonhuman primates, wild mammalians and ratite birds [4], [5]. Outbreaks among foals began to be reported on breeding farms worldwide within the last decade [6], [7]. Therefore, PE is now considered an emerging disease in horses [8].

Although PE was first reported in 1931 [9], the causative bacterium was primarily isolated only in 1993 using rat small intestinal cells (IEC-18) in strict microaerophilic environmental conditions [10]. Since then, various cell lines have supported *L.* *intracellularis* growth *in vitro*, including insect and avian cell lines [11]–[14]. To date, growth of the bacteria in axenic (cell-free) media has not been reported. Regardless of the cell type, the dynamics of the infection *in vitro* requires actively dividing cells in a microaerophilic atmosphere with the peak of infection at six to seven days post-inoculation [10], [14], [15]. McOrist et al (1995) chronologically described the dynamics of the infection and bacterial replication in intestinal porcine epithelial cells (IPEC-J2). Most events closely resembled those observed at the cellular level in infected animals, including multiplication of the bacteria freely in the cell cytoplasm. While the dynamics of the infection have been well-characterized, little is known so far about the genetic basis for the virulence, pathogenesis or physiology of *L. intracellularis* [16].

Spontaneous attenuated isolates obtained through multiple passages in cell culture have not been successful at inducing typical PE lesions or reversing its virulence in experimentally-infected pigs [12]. Conversely, bacterial isolates at low passage induce clinical and pathological changes typical of PE [17]. Various standard DNA-based typing techniques, such as pulsed field gel electrophoresis (PFGE), multilocus sequence typing (MLST) and variable number tandem repeat (VNTR) have shown identical genotypes in both pathogenic (low passage) and non-pathogenic (high passage) variants [18]–[20]. As a result, we believe their phenotypic properties occur at the transcriptional level. Bacterial genes differentially expressed between pathogenic (low passage) and non-pathogenic (high passage) homologous isolates have not been reported and this information may help to elucidate genes encoding the major bacterial virulence factors involved in the pathogenesis of PE.

We hypothesize that genes differentially expressed between pathogenic (passage 10) and non-pathogenic (passage 60) homologous *L. intracellularis* isolates encode potential bacterial virulence factors. High-throughput technology (RNA-seq) was used to characterize and compare the transcriptional profile of a pathogenic and a non-pathogenic variant. Plasmid-encoded genes, regulatory factors and ATP-binding cassette (ABC) transporters associated genes were important for contributing to the wider transcriptional landscape observed in the pathogenic isolate. Additionally, the present study provided novel information for studying specific mechanisms of target genes and their potential usefulness for the diagnosis and control of PE.

## Results and Discussion

Whole-transcriptome profiling of bacterial organisms has been widely studied to understand global changes in gene expression *in vitro* and *in vivo* [21], [22]. Hybridization-based approaches have been successfully applied for years [23], but it has limited use in obligate intracellular bacteria since it needs a reference purified sample from cultivated bacteria. High-throughput technologies have overcome these limitations by providing high resolution data to describe the bacterial transcripts in different experimental conditions [24], [25]. The present study used RNA-seq to qualitatively and quantitatively characterize the transcriptional profile of pathogenic and non-pathogenic homologous *L. intracellularis* isolates during *in vitro* infection. Since this is the first comprehensive gene expression analysis regarding this organism, the findings are presented and discussed in a comparative pathogenomic approach based on the information available from other related bacterial organisms.

### Mapping and Differential Expression

The sequence reads representing the RNA transcripts derived from the pathogenic and non-pathogenic homologous *L. intracellularis* PHE/MN1-00 isolates were mapped onto the complete genome sequence of the same bacterial strain available at the National Center for Biotechnology Information (NCBI) (accession: NC_008011). The circular *L. intracellularis* genome has 1,719,014 base pairs (bp) comprised of one chromosome (1,457,619 bp) and three plasmids (plasmid A: 27,048 bp, plasmid B: 39,794 bp and plasmid C: 194,553 bp). From a total of 1,391 computationally predicted genes in the annotated PHE/MN1-00 genome, 1,340 are protein coding. Combining the pathogenic and non-pathogenic transcript reads, 731 protein coding genes were mapped onto the reference DNA sequence of the *L. intracellularis* PHE/MN1-00 isolate. Sequence reads mapped against the bacterial genome were used to quantify the gene expression levels based on the number of reads per kilobase of coding sequence per million mapped reads (RPKM). The expression data were sufficiently reproducible by showing a positive correlation between the two biological replicates of the pathogenic (r^2^ = 0.86) and non-pathogenic (r^2^ = 0.81) variants which was obtained using a linear regression model (*p*<0.05). Genes with low expression levels showed lesser agreement between replicates (Figure S1). This observation was reported using Illumina® RNA-seq data from human brain RNA [26] and using ABI SOLiD™ platform in a transcriptome study of *Neisseria gonorrhoeae* [25]. Following the default parameters of the CuffDiff software (see Material and Methods) our study used ten sequenced reads as the minimum number of alignments in a locus needed to conduct agreement and comparison testing within and between the biological replicates.

**Figure 1:**
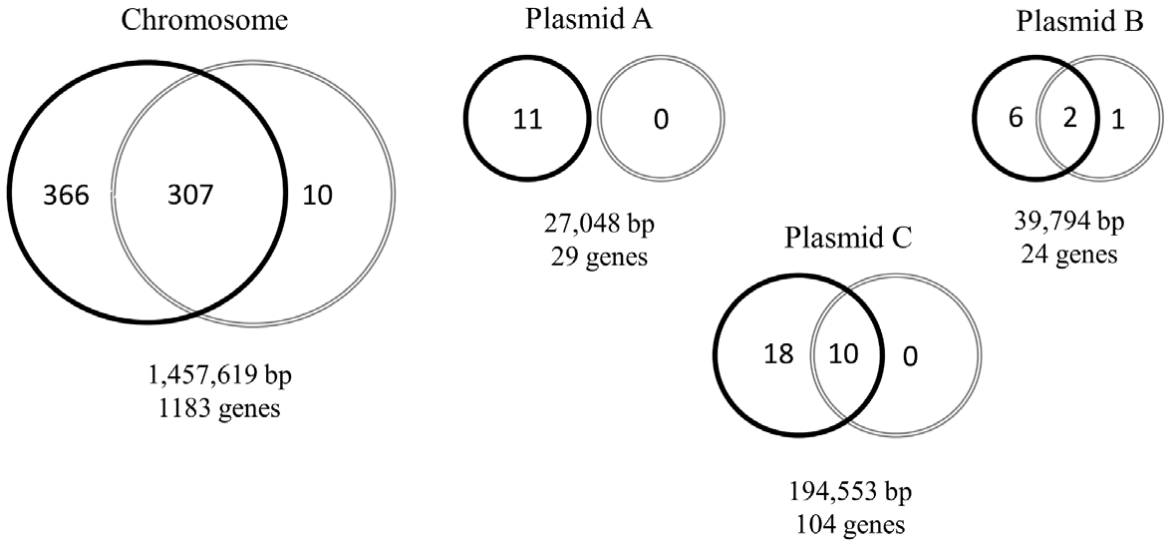
Schematic representation of the *L. intracellularis* genome. Distribution of genes expressed by the pathogenic (black circles) and the non-pathogenic (gray circles) variants. Overlapping zones represent genes expressed in both variants.

A total of 720 and 330 genes were expressed by the pathogenic and non-pathogenic variants, respectively (Figure S2). The wider transcriptional landscape observed in the pathogenic variant was characterized by 401 genes uniquely expressed by this variant. Genes with the highest transcription levels according to their biological function categories are shown in Table 1. Only 11 genes were expressed exclusively by the non-pathogenic variant (Table 2). Differential mapping and distribution of the expressed genes into the *L. intracellularis* chromosome and its three plasmids are summarized in Figure 1. Plasmid-encoded genes significantly contributed to this broader profile of gene expression exhibited by the pathogenic isolate. The entire transcriptional repertoire of the plasmid A was suppressed in the non-pathogenic isolate suggesting its potential contribution in the *L. intracellularis* virulence. Transcription factors located in bacterial chromosomes have been shown to positively regulate virulence factors in the plasmid of *Shigella flexneri* and *Pseudomonas syringae* [27], [28]. Our study also identified many regulatory factors exclusively expressed by the pathogenic *L. intracellularis* variant which potentially regulate plasmid-encoded genes. Specific findings related to regulatory factors are discussed later.

**Table 1:** Chromosomal genes showing highest transcript levels exclusively expressed by the pathogenic variant of the *L.* *intracellularis* isolate PHE/MN1-00 according to the putative biological function.

Locus	Gene	Description	Log_2_ (RPKM)
*Biological function*			
*Cell division and macromolecule biosynthesis*			
LI0372		putative cell division protein *FtsB*	12.8
LI0844		tRNA and rRNA cytosine-C5-methylases	14.1
LI0962	*rplW*	50S ribosomal protein L23	13.5
*Energy production and conversion*			
LI0442	*hyaD*	processing of HyaA and HyaB proteins	10.9
LI1176		nitroreductase	11.1
*Cellular processes and small molecule biosynthesis*			
LI0154	*ribH*	riboflavin synthase beta-chain	12.6
LI0361		quinolinate synthetase	12.7
LI0735	*ychB*	4-diphosphocytidyl-2-C-methyl-D-erythritol kinase	14.5
*Membrane transport*			
LI0338	*livH*	branched chain amino acid ABC transporter (permease)	12.5
LI0754	*glnH*	amino acid ABC transporter substrate-binding protein	13.9
LI0995	*oprM*	outer membrane efflux protein	12.1
*Protein turnover and chaperones*			
LI0375	*pcm*	protein-L-isoaspartate carboxylmethyltransferase	12.2
LI0225	*smpB*	SsrA-binding protein	11.4
LI0618		ATPases with chaperone activity, ATP-binding subunit ClpA	11.4
*Cell membrane and motility*			
LI0825		Lipid A core-O-antigen ligase and related enzymes	13.2
LI0947		cell wall-associated hydrolases	12.6
LI1141	*cheW*	chemotaxis signal transduction protein	11.0
*Adaptation and stress response*			
LI0035	*fur*	Fe2+/Zn2+ uptake regulation proteins	11.8
LI0301	*recO*	DNA repair protein RecO (recombination protein O)	13.0
LI0457	*rpoN*	Sigma54-like protein	11.7
*General predicted function*			
LI0140		glycosyltransferase	11.5
LI0246	*hypA*	zinc finger protein	11.6
LI0880	*sfsA*	DNA-binding protein, stimulates sugar fermentation	11.4
*Hypothetical/Unknown function*			
LI0259		hypothetical protein	13.9
LI0917		hypothetical protein	12.9
LI1056		hypothetical protein	12.3

**Table 2:** Genes exclusively expressed by the non-pathogenic variant of the *L. intracellularis* isolate PHE/MN1-00.

Locus	Gene	Description	Log_2_ (RPKM)
*Biological function*			
*Cell division and macromolecule biosynthesis*			
LI0702	*maf*	nucleotide-binding protein involved in septum formation	11.7
LIB024	*parA*	ATPases involved in chromosome partitioning	11.1
*Energy production and conversion*			
LI0614		Thiol-disulfide isomerase and thioredoxins	12.1
LI1001	*napC*	cytochrome c nitrite reductase small subunit	12.2
*Cellular processes and small molecule biosynthesis*			
LI0709	*ribF*	FAD synthase involved in riboflavin metabolism	12.2
*Membrane transport*			
LI0540	*yscN*	type III secretion system ATPase	11.3
LI1163	*YscJ*	type III secretion protein	10.8
*Cell membrane and motility*			
LI0747	*flgK*	flagellar hook-associated protein	11.2
*Predicted and unknown function*			
LI0427		hypothetical protein	9.8
LI0554		hypothetical protein	10.8
LI0583		hypothetical protein	10.1

A correlation between infectivity and loss of DNA contents from linear and/or circular plasmids during serial passages *in vitro* has been well established in *Borrelia burgdorferi* infections [29], [30]. Since there are a large number of different plasmids found in *Borreliae* genomes, their stability and loss *in vitro* vary between and within species [31]. Although our study did not identify any transcriptional activities in the plasmid A of the non-pathogenic variant, the DNA contents of this plasmid were identical in both pathogenic and non-pathogenic variants (data not shown). Additionally, transcription levels were detected in three and ten genes present in the plasmid B and C of the non-pathogenic variant, respectively (Figure 1). These findings demonstrated the occurrence of transcriptional activities and, consequently, the presence of these two plasmids in this variant. These observations support the hypothesis that global changes in gene expression are able to drive the loss of the virulence phenotype in cultivated *L. intracellularis* without altering the genomic DNA.

**Figure 2:**
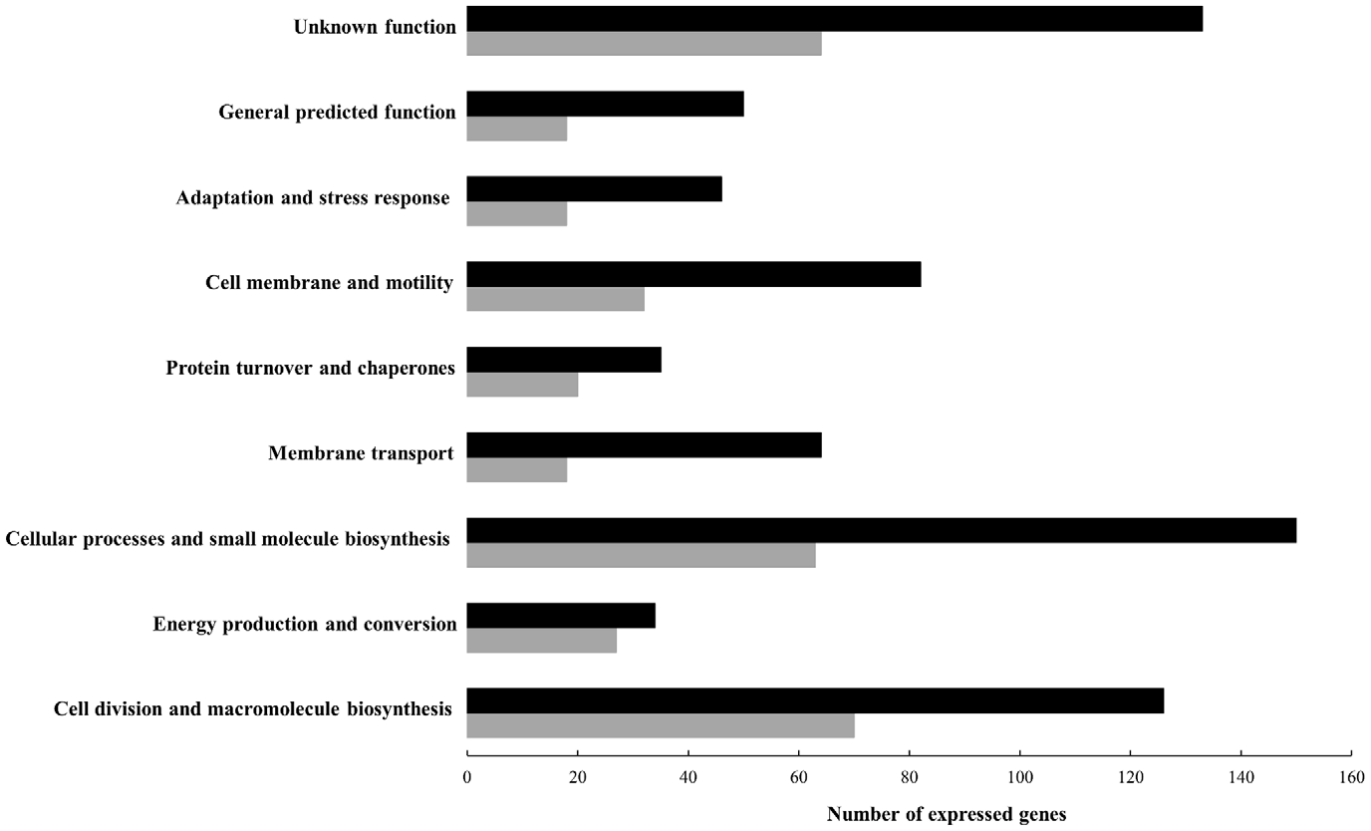
Functional categories of genes expressed by the pathogenic and non-pathogenic homologous *L. intracellularis* isolate PHE/MN1-00. Black and gray bars represent the number of genes expressed by the pathogenic and non-pathogenic variants, respectively.

**Figure 3:**
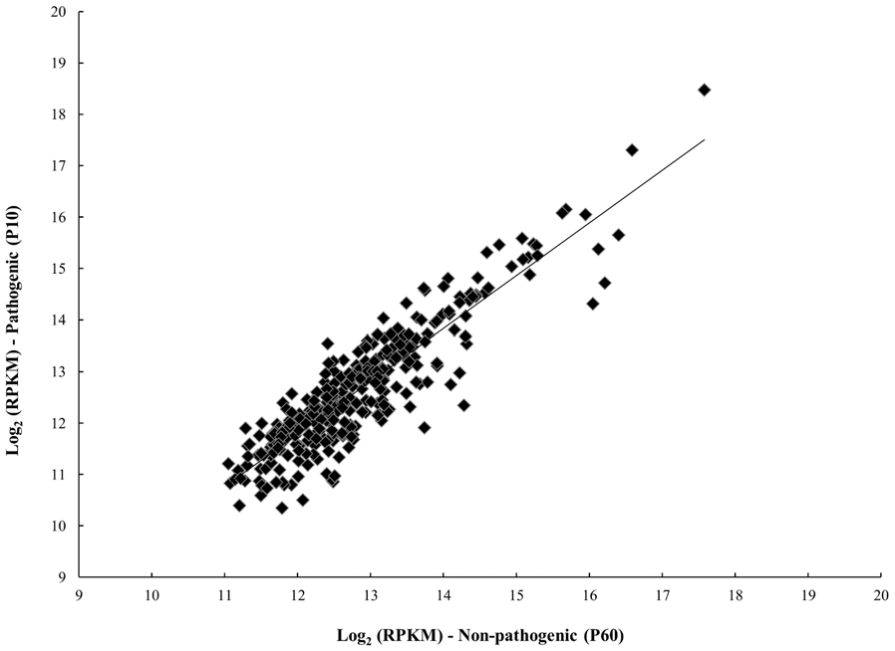
Average log-transformed RPKM (log_2_ [RPKM]) of the 319 genes commonly expressed by the pathogenic (y-axis) and non-pathogenic (x-axis) variant. The trend line represent a linear regression model (*p*-value <0.05; r^2^ = 0.809).

Narrowing the analysis to the putative biological gene functions, the number of genes expressed by the pathogenic variant was consistently higher throughout the functional categories (Figure 2). The transcriptional landscape was most significantly reduced in those genes involved in the membrane transport (72%), general predicted function (64%), cell membrane and motility (61%) and adaptation and stress response (61%) categories. In comparing the transcription levels of all 319 genes commonly expressed in both the pathogenic and non-pathogenic variants, there was no significant difference (fold change ±2; adjusted *p*-value <0.05– Table S1). When the expression levels from both variants were plotted against each other (Figure 3), log_2_ (RPKM) values unexpectedly showed a positive correlation (0.809) in a linear regression model. These results revealed the importance of gene silencing (switching off) mechanisms to attenuate virulence properties of *L. intracellularis* throughout passages *in vitro*.

Regulation of gene expression during *in vitro* cultivation has been widely reported in various bacterial organisms [32]. *Bordetella pertussis* switches off the expression of type III secretion system (TTSS) proteins in laboratory-adapted strains [33]. One of the TTSS components, protein Bsp22, ceases to be expressed between passages three and four [34]. Interestingly, these authors also showed the reversible re-expression of this protein after contact with the host *in vivo*. The expression of TTSS proteins has been demonstrated during *L. intracellularis* infection [35]. However, our study failed to demonstrate consistent differences in the expression of TTSS proteins between pathogenic and non-pathogenic variants. Additionally, reversibility of virulence has not been reported in animals infected with laboratory-adapted *L. intracellularis*.

Transcriptional regulation has also been associated with mutations characterized by non-synonymous substitutions at the DNA level in *Escherichia coli* cultivated in glucose-limited medium for 20,000 generations [36]. Regulatory changes in gene expression following by mutations in 12 lines of *E. coli* were driven by the specific environment *in vitro* evolving an ecological specialization in laboratory-adapted strains [37]. Narrower transcriptional profiling of the non-pathogenic *L. intracellularis* passed 60 times *in vitro* was observed in our study; this could represent earlier stages of adaptation to a specialized *in vitro* environment. As in the case of *E. coli*, unnecessary functions that are costly to fitness in the *in vitro* condition might then be eliminated after hundreds or thousands of generations. According to ecological specialization models, organisms genetically adapted to one environment may lose fitness in other environments [37], [38]. Corroborating with this principle, the lack of reversible virulence in animals infected with laboratory-adapted *L. intracellularis* suggests that improving fitness in a specialized environment *in vitro* adversely affects performance in other complex substrates *in vivo*.

**Figure 4:**
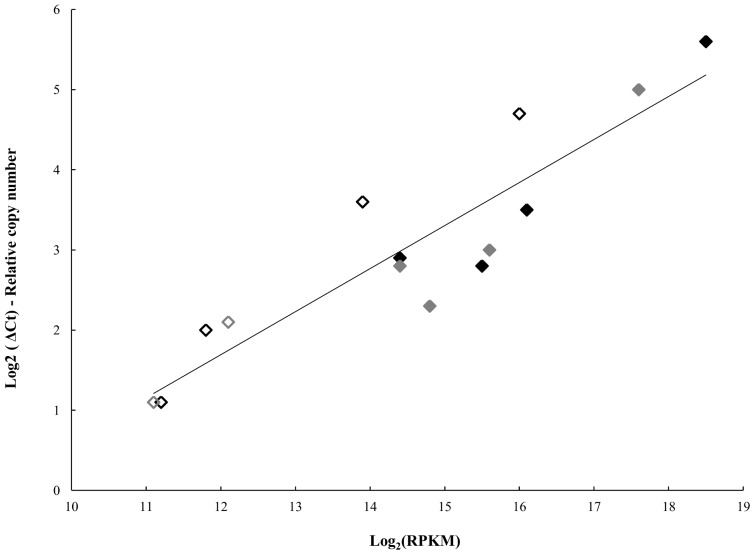
Correlation between RNA-seq and qRT-PCR. Plot demonstrating the relative quantification of 10 unlinked genes by qRT-PCR (y-axis) and the transcript levels generated by RNA-seq (x-axis). Genes commonly expressed by the pathogenic (♦) and non-pathogenic (♦) variants. Genes exclusively expressed by the pathogenic (**◊**) and non-pathogenic (**◊**) variants.

Gene expression data generated by RNA-seq were validated using quantitative reverse transcriptase PCR (qRT-PCR). The reliability of RNA-seq results was confirmed based on the relative quantification of 10 unlinked genes: four expressed in both variants, four expressed by the pathogenic and two expressed by the non-pathogenic (see Materials and Methods). The average log-transformed from two qRT-PCR replicates was plotted against the log_2_ (RPKM) (Figure 4). The transcript levels were positively correlated on a linear regression model (*p*-value <0.05; r^2^ = 0.827). Therefore, the RNA-seq data were consistent in quantitatively estimating the transcription levels of *L. intracellularis* during infection *in vitro*. The validation of RNA-seq data using qRT-PCR has also been previously reported in yeast [39] and other bacterial transcriptomes [24], [25], [40].

### Cell Division and Macromolecule Biosynthesis

Synthesis of proteins involved in cell division and ribosome biogenesis has been closely related to bacterial growth rate [41], [42]. From a total of 43 ribosomal protein-encoding genes present in the *L. intracellularis* reference genome, we identified 31 genes expressed by both the pathogenic and non-pathogenic variants and eight additional genes exclusively expressed by the pathogenic one. Furthermore, the operon responsible for orchestrating bacterial cell division that contained the *FtsA* and *FtsZ* genes [43] was also identified in both variants (Table S1). The expression of RNA polymerase á and â subunits has also been associated with growth rate in that their synthesis increases in response to a rich nutrient medium (nutritional shift-up) [44]. All *L. intracellularis* genes encoding RNA polymerase subunits previously predicted from the annotated genome were expressed in both variants. Regardless of the pathogenic or non-pathogenic phenotype, these observations demonstrated that both variants grew at the same rate and were harvested during exponential phase at time of RNA harvesting (five days post-infection). Likewise, the results corroborate previous studies which described an increase in the number of *Lawsonia*-infected cells from two to seven days after infection [15], [45]. Adverse effects in bacterial growth have been related to the reduced expression of RNA polymerase subunits and ribosomal proteins in *Neisseria gonorrhoeae* cultivated in an oxygen-limited environment [25]. The microaerophilic environment provided in our study was first described by Lawson et al (1993) and it has been shown to be an optimal atmosphere for cultivation of *L. intracellularis* [10], [45].

### Energy Production and Conversion

Pathogenic bacteria are frequently able to couple virulence pathways with general metabolic functions such as energy production, cellular signaling and molecular biosynthesis [46]. The operon comprising F_0_–F_1_ ATP synthase subunits, which was expressed in both pathogenic and non-pathogenic *L. intracellularis* variants (Table S1), is essential for life in many bacterial species since it maintains pH homeostasis of the cell [47]. Concomitantly, it plays a critical role in the acid stress response, specifically in enteric bacteria which encounter low pH conditions transiting through stomach and in the phagosome of host cells [48]. Although we intended to harvest *L. intracellularis* freely multiplying in the cell cytoplasm (five days post-infection) which would fully represent its exponential growth phase, the identification of the F_0_–F_1_ operon in both variants suggests that a fraction of harvested intracellular bacteria was in the transient phagosome phase.

Along with these observations, the expression data also indicated the presence of free intracellular bacteria in the cell cytoplasm by detecting high transcriptional levels (Table S1) of rickettsia-like ATP/ADP translocase gene in both variants. This transmembrane protein was first identified in a few obligate intracellular bacteria, including *Chlamydiales*, *Rickettsiales* and amoebal symbionts [49], and more recently in *L. intracellularis* [50]. A remarkable adaptation of these organisms to an intracellular microenvironment allows them to import cytoplasmic ATP generated in their hosts into the prokaryotic cell across the bacterial cell membrane [51]. In an exchange mode, the bacterial ADP is exported back into the host cytosol. This exploitation of the host’s energy pool is also referred to as energy parasitism. In agreement with the exponential growth phase of *L. intracellularis*, we also identified the expression of cytochrome *bd* operon in the pathogenic and non-pathogenic variant (Table S1). This oxygen reductase is used in the bioenergetic pathway in a variety of bacterial organisms under O_2_-limited conditions [52].

### Cellular Processes and Small Molecule Biosynthesis

Although *L. intracellularis* and other obligate intracellular bacteria depend on their hosts for certain nutrients (e.g. cytoplasmic ATP, see above), a wide spectrum of biosynthetic pathway-encoding genes are critical for fulfilling their essential functions. The 2-C-methyl-D-erythritol 4-phosphate (MEP) pathway is primarily involved in the biosynthesis of structural and functional isoprenoid molecules but the genes encoding the final two enzymes, *ispG* and *lytB*, have also been related to intracellular survival and induction of cellular immune response [53]. Six genes of the *Lawsonia* MEP pathway previously predicted in the Kyoto encyclopedia of gene and genomes (KEGG) database were exclusively expressed by the pathogenic variant. However, the final remaining two enzymes (*ispG* and *lytB*) were transcribed by both the pathogenic and non-pathogenic variants. We suggest that *ispG* and *lytB* may be essential genes in *L. intracellularis*, as previously reported in several other bacterial pathogens [53]. Based on this vital characteristic of the MEP pathway, it has recently been used as a drug target [54]. Fosmidomicyn showed the ability to inhibit the second enzyme of the MEP pathway (*Dxr*) and has been used in clinical trials [55]. Interestingly, our study identified expression levels of the *Dxr*-encoding gene (log_2_ [RPKM]  = 10.1) exclusively in the pathogenic variant, suggesting its potential use for drug targeting.

Despite the similarities in the expression of ribosomal proteins and RNA polymerase subunits between pathogenic and non-pathogenic variants described previously, our study also identified the expression of *relA* gene exclusively in the pathogenic variant. This gene encodes an enzyme responsible for synthesizing guanosine tetraphosphate (ppGpp). This small signal molecule, also called stringent factor, is involved in various cellular processes and affects the control of growth rates by binding to RNA polymerase and reduces synthesis of ribosomal proteins and tRNA molecules during nutritional stress [56], [57]. Although the transcript reads of *relA* gene were exclusively identified in the pathogenic variant, its expression levels were relatively low (log_2_ [RPKM]  = 9.7) suggesting that it was not sufficient to affect the expression of genes involved in macromolecule biosynthesis.

Based on the empirical observation that high-passage variants of *L. intracellularis* have higher growth rates than low passages *in vitro*, we believe that *relA*-dependent accumulation of ppGpp may control the bacterial growth rate at some point during the course of the infection *in vitro*. However, chronological transcriptional analysis is needed in order to elucidate this question. Supporting this speculation, Cooper et al (2003) demonstrated an increased fitness of laboratory-adapted *E. coli* (after 20,000 generations *in vitro*) associated with reduction in the concentration of ppGpp. These authors did not observe any mutation in the DNA sequence of *relA* gene. Similarly, the suppression of *Lawsonia relA* gene in the non-pathogenic variant was not associated with any mutations in the *relA* DNA sequence (data not shown).

The stringent response mediated by ppGpp also plays an important role in bacterial virulence, especially to adapt to conditions encountered in the intracellular host environment [46]. A *relA* mutant strain of *Listeria monocytogenes* showed no virulence *in vivo* using murine infection models [58]. However, the same study shows no difference between the wild type and Ä*relA* mutant strain in their ability to escape from the phagosome and polymerize host cell actin *in vitro* using Caco-2 cells. Comparable phenotypic differences between *in vitro* and *in vivo* infections have also been observed in *L. intracellularis*. Low passage and high passage variants have similar abilities to infect cells *in vitro* but they demonstrated differences in pathogenic and non-pathogenic phenotypes *in vivo* [59].

### Membrane Transport

The majority of genes encoding ABC transporters identified in the *L. intracellularis* genome were shown to be expressed in the pathogenic variant. From a total of 14 ABC transporter operons, 10 cassettes were specifically expressed by the pathogenic variant, including those involved in resistance to organic solvents, membrane transport of polyamines (spermidine), phosphates, nitrates, amino acids (glutamine and branched-chain), metallic cations (cobalt and nickel), lipopolysaccharides and lipoproteins. We specifically observed the highest expression levels in ABC uptake systems for amino acids (Table 1). Elevated transcription levels of the glutamine transport system (*glnHPQ* operon) were shown to be essential for virulence in *Salmonella enterica* Serovar Typhimurium [60] and *Streptococcus pneumoniae* [61]. Both studies demonstrated significant attenuation of the virulence *in vivo* using mouse models infected with mutant strains (Ä*glnHPQ*). Despite the attenuated phenotype, the mutant strain of *S. enterica* Typhimurium provided a protective immune response against challenge with wild-type. Additionally, the authors showed that *glnHPQ* operon is positively regulated by the sigma factor ó^54^ (RpoN). This regulatory factor was also exclusively expressed by the pathogenic *L. intracellularis* variant in our study (Table 1). The expression of ó^54^ and other transcription factors are discussed later in the subsection related to bacterial adaptation and stress response.

The *in vitro* growth kinetics of *S.* *enterica* Typhimurium and *S. pneumoniae glnHPQ* mutants were not affected in either broth or agar supplemented with glutamine or peptides [60], [61]. On the other hand, both studies showed reduced intracellular survival of these mutants in macrophage cell lines. There is no information regarding the intracellular survival of pathogenic or non-pathogenic *L. intracellularis* in infected macrophages and no impairment of intracellular survival has been reported in *L. intracellularis* continually cultivated using epithelial or fibroblastic-like cells [15], [45]. Although the IPEC-J2 cell culture media used in our study was not supplemented with glutamine, the glutamine ABC transporters were consistently expressed only by the pathogenic variant in the two *glnHPQ* operons present in the *L. intracellularis* genome.

The ABC transporter involving polyamine uptake (*PotABCD* operon) and uniquely expressed in the pathogenic variant at moderate levels (log_2_ [RPKM]  = 11.1) has also been important for virulence in other bacterial organisms [62]. Corresponding with the glutamine ABC transporters discussed above, mutations in the *PotABCD* operon of *S. pneumoniae* showed no effect on the growth rates *in vitro*, but the mutant strain showed significant attenuation of virulence within murine models regardless of the inoculation route [62].

The differential mapping of other bacterial transport systems including TTSS and general secretory (Sec) pathways was not consistently different between the pathogenic and non-pathogenic variants. The role of TTSS proteins has been well established at earlier stages of infection and has been required for the invasion of various bacterial organisms [63]. Our study detected the intracellular expression of five of a total of 15 *L. intracellularis* genes involved in the synthesis of TTSS previously identified in the KEGG database. This unexpected intracellular expression of TTSS proteins was reported in *S. enterica* Typhimurium and plays a role in the survival of this organism within the *Salmonella*-containing vacuole [64], [65].

The expression of genes encoding structural components of TTSS (*YscN*, *YscO* and *YscQ*) was previously identified by RT-PCR in three *L. intracellularis* isolates infecting rat small intestinal cells (IEC-18) [35]. However, the study did not provide information about the number of cell passages the isolates had undergone or the time points in which the bacterial RNA was harvested from the infected cell monolayers. Our study identified two (*YscQ* and *YscN*) of the three major TTSS components cited above. The *YscQ* gene was expressed in both the pathogenic and non-pathogenic variant and *YscN* was uniquely identified in the non-pathogenic variant (Table 2). These variable results regarding the expression of TTSS proteins suggest that further studies are required in order to elucidate the role of TTSS in *L. intracellularis* infection *in vitro*.

### Protein Turnover and Chaperones

Chaperone-encoded genes (*DnaK, DnaJ, GroEL and GroES*) were highly expressed by the pathogenic and non-pathogenic variants. This observation corroborates the up-regulation of this gene category identified in a variety of bacterial organisms growing inside eukaryotic cells [66]. Furthermore, bacterial chaperones are known to be essential for overcoming bacterial stress by ensuring the proper folding of proteins. Three additional genes (*CbpA*, *CrpE* and *ClpA*) involving the synthesis of chaperone molecules were exclusively expressed by the pathogenic variant and their specific biological functions remain to be determined.

### Cell Membrane and Motility

A broader diversity of genes encoding cell wall molecules were observed in the pathogenic *L. intracellularis* variant, suggesting more extensive options for remodeling of the bacterial envelope compared with the non-pathogenic variant. This characteristic is important for altering the bacterial cell surface during different steps of the infectious process [66]. The wider variety of this gene category in the pathogenic variant was predominantly comprised of genes encoding glycosyltransferase enzymes which have been implicated in posttranslational processing of proteins responsible for modifying the lipopolysaccharide composition. Modifications of the glycosylation composition of the cell wall were depicted in *Campylobacter jejuni* during its intestinal life cycle. These events were then considered part of the strategy used by the bacterium to resist and evade the host immune responses [67].

In the mapping of genes involved in the flagellar assembly pathway, there were no consistent differences between the pathogenic and non-pathogenic variants. From a total of 30 predicted genes in the KEGG database, ten were expressed in both variants (Table S1), eight were exclusively identified in the pathogenic and one was unique from the non-pathogenic variant (Table 2). Our study detected similar expression levels of the *FliC* gene in both variants (Table S1). The flagellin encoded by this gene has been well studied regarding its ability to induce an immune response [68]. *S. enterica* Typhimurium expressed this protein in the membrane-bound compartment and it was then translocated into the host cytoplasm to be detected by cytosolic Nod-like receptors which are able to mount an innate immune response [69]. Studies characterizing the immune response of cells infected with pathogenic and non-pathogenic *L. intracellularis* would be important to detect specific host factors associated with these two variants.

### Adaptation and Stress Response

The number of genes encoding proteins involved in adaptation and stress response was one of the gene categories with significant reduction (61%) in the non-pathogenic variant (Figure 2). Ten of the 28 genes uniquely expressed by the pathogenic variant in this category were transcriptional regulatory factors. These molecules regulate bacterial gene expression by acting globally or at specific DNA regions to active or repress transcription or modulate DNA topology. The ó^70^ is the “housekeeping” sigma factor and has been required for cell growth in the majority of bacterial organisms [70]. In agreement with this, we observed similar expression levels of ó^70^ in the pathogenic and non-pathogenic variants (Table S1). Conversely, the global regulator ó^54^ was uniquely expressed in the pathogenic variant (Table 1) and has been reported to control the transcription of virulence genes in a variety of bacteria species [70]. Specifically in *S. enterica* Typhimurium, the role of ó^54^ is to coordinate the transcription of the glutamine ABC transporter, *glnHPQ* operon, during the intracellular life cycle. The coincidental and exclusive co-expression of ó^54^ and *glnHPQ* by the pathogenic variant supports the hypothesis that this sigma factor may also coordinate the glutamine uptake operon in *L. intracellularis* to ensure an adequate supply of this crucial amino acid during its intracellular life. Additionally, the consistently higher number of ABC transporter-encoding genes expressed by the pathogenic, but not the non-pathogenic, variant begs the question whether ó^54^ is also able to positively regulate other ABC transporters (e.g. polyamines and branched-chain amino acid transporters). The obligate intracellular nature of *L. intracellularis* has imposed considerable limitations in elucidating this and other specific regulatory mechanisms. To date, the construction of recombinant plasmids appears to be an alternative model for overcoming these barriers.

A gene encoding the major transcription factor involved in the iron homeostasis (*fur* regulator) was uniquely expressed by the pathogenic variant (Table 1) and its transcription levels were confirmed by qRT-PCR (Figure 4; Table S2). The *fur* regulator can act as either a repressor or an activator in a variety of bacterial organisms [71]. The wider gene expression profile identified in the pathogenic variant of *L. intracellularis* suggests the role of *fur* as an activator in our experimental conditions. Supporting this hypothesis, *fur*-activated genes have been reported in other enteric pathogens (e.g. *S. enterica* Typhimurium) and bacteria that replicate freely in the host cytoplasm (e.g. *L. monocytogenes*) [71], [72]. Although this transcription factor is typically related to the iron metabolism in response to iron availability, its role in the stress response and virulence *in vivo* has also been well established [71]. For instance, *fur* mutants of *L. monocytogenes* and *C. jejuni* showed reduced virulence in experimental models [73], [74]. Furthermore, the *in vitro* growth rates in a *fur* mutant strain of *Desulfovibrio vulgaris*, one of the closest bacterial species genetically related to *L. intracellularis* [75], [76], were not affected.

In addition to its role in virulence, *fur* acts in the regulation of genes involved in oxidative stress, such as superoxide dismutase (*sod*). However, the unique *sod* gene previously annotated in the *L.* *intracellularis* genome (*sodC* gene) was expressed at high levels by both pathogenic and non-pathogenic variants (Table S1). Its transcription levels were validated by qRT-PCR (Figure 4; Table S2). This finding indicates that a *fur*-independent mechanism potentially regulates *sodC* expression *in vitro*. In agreement with this speculation, the literature describes *fur* regulation of *sodA* (Mn^2+^-containing *sod*) and *sodB* (Fe^2+^-containing *sod*) but not *sodC* (Cu-Zn^2+^-containing *sod*) [71]. In addition, *fur* mutation had no effect on the regulation of *sodC* in *E. coli* [77]. Regardless of specific regulatory mechanisms, the expression of the *sodC* gene is critical for intracellular survival of pathogenic bacteria by catalyzing reactive host-derived superoxide radicals (O_2_^−^) to hydrogen peroxide (H_2_O_2_) and oxygen (O_2_) [78]. In our study, this mechanism also appears to be essential for both pathogenic and non-pathogenic *L. intracellularis* during *in vitro* infection. The gene (*rubA*) encoding rubredoxin 2 (Rb-2) was computationally predicted in the *L. intracellularis* genome and has been described to complement the catalytic activity of *sodC* against oxidative stress in *Desulfovibrio* species. Our study identified *rubA* as the second most commonly expressed gene by both pathogenic and non-pathogenic variants (Table S1). According to a model proposed by Lumppio et al (2001), the superoxide dismutase acts in the periplasm fraction and the Rb-2 neutralizes superoxide radicals in the bacterial cytoplasm [79].

### Predicted/unknown Function

Genes encoding hypothetical proteins comprise approximately 27.2% of the 1340 protein encoding genes previously predicted in the reference DNA sequence of the *L. intracellularis* PHE/MN1-00 isolate. This significant number becomes more evident within the plasmid A (55.2%), plasmid B (58.3%) and plasmid C (63.5%). Our study identified four genes encoding hypothetical proteins in the ten most highly expressed genes commonly identified in the pathogenic and non-pathogenic variants. Among all commonly expressed genes, the LI0447 gene demonstrated the highest transcription levels (Table S1) which was confirmed by qRT-PCR (Figure 4; Table S2). The protein encoded by the LI0447 gene was computationally predicted to be a transmembrane protein with 50.7% identity at the amino acid level to other hypothetical proteins identified in the *Mannheimia haemolytica* genome (MHA_1476– accession: ZP_04978003.1). An autotransporter protein (LatA) was recently characterized as a prominent antigen during infection of IEC-18 cells with the *L. intracellularis* isolate LR189/5/83 [80]. However, the authors did not specify the number of *in vitro* passages that bacterial isolate had. Our study identified the gene encoding this protein, referred to as LI0649, in both the pathogenic and non-pathogenic variants (Table S1). Regarding the genes uniquely expressed by the pathogenic variant, the hypothetical protein encoded by the LI0259 gene was the third most highly expressed chromosomal gene (Table 1). This protein (accession: YP_594636) has a conserved putative domain of 41 amino acids found in a wide range of bacteria and is noted as a regulatory factor included in the FmdB family.

From a total of 35 plasmid-encoded genes uniquely expressed by the pathogenic variant (Figure 1), 19 genes were predicted to encode hypothetical proteins. Genes showing the highest transcription levels in the plasmids are summarized in Table 3. The LIC056 gene was the most highly expressed gene identified uniquely in the pathogenic variant and its transcription levels were confirmed by qRT-PCR (Figure 4; Table S2). This gene encodes a predicted transmembrane protein containing a conserved autotransporter beta-domain. This class of proteins has been implied to mediate secretion by translocation of bacterial proteins across the outer membrane. Another intriguing observation was the expression of the LIC091 gene exclusively in the pathogenic variant (Table 3). This gene has been predicted to encode one of the largest proteins among all bacterial organisms containing 8746 amino acids. Eight different protein families have been proposed for this molecule including a transmembrane protein with 99.5% of its amino acid sequence belonging to an extracellular domain. Our study provides evidence regarding the potential role of genes encoding hypothetical proteins during *L. intracellularis* infection *in vitro*, but their specific biological functions remain to be elucidated. Additionally, the substantial number of these genes previously identified in the *L. intracellularis* genome and now associated with its transcriptional profiling suggests that this organism may adopt unique mechanisms of survival and pathogenesis among bacterial pathogens.

**Table 3:** Plasmid-encoded genes with highest transcript levels exclusively expressed by the pathogenic variant of the *L.* *intracellularis* isolate PHE/MN1-00.

Locus	Gene	Description	Log_2_ (RPKM)
*Biological function*			
*Plasmid A*			
LIA022		cell wall biosynthesis glycosyltransferase	12.1
LIA025		plasmid stabilization system protein	11.7
LIA017	*bchE*	Fe-S oxidoreductase	11.2
*Plasmid B*			
LIB002		PbsX family transcriptional regulator	13.5
LIB017		hypothetical protein	12.1
LIB019		hypothetical protein	13.3
*Plasmid C*			
LIC047		hypothetical protein	12.8
LIC056		hypothetical protein	16
LIC079		hypothetical protein	11.4
LIC091		hypothetical protein	11.4

### Conclusions

The present study is the first report characterizing the transcriptional profile of *L. intracellularis* and comparing the gene expression levels of a pathogenic and a non-pathogenic homologous isolate. The wider transcriptional landscape identified in the pathogenic variant was consistent throughout the gene categories and had significant contributions of plasmid-encoded genes. High expression levels of genes encoding ABC transporters and specific transcriptional regulators were uniquely identified in the pathogenic variant and suggest specific metabolic adaptation of *L.* *intracellularis*, including substrate acquisition that allows its efficient proliferation in the infected host. The dynamics of the genetic changes in laboratory-adapted bacterial organisms have developed a new ecological specialization which results in different bacterial phenotypes [37]. In our study, the lack of selective pressure during multiple cell passages *in vitro* might be the reason for the narrower transcriptional profile observed in the non-pathogenic variant and loss of pathogenicity *in vivo* by gene silencing (switching off) mechanisms.

The diversity of genes exclusively expressed in the pathogenic variant and repressed in the non-pathogenic, including those involved in membrane transport, adaptation and stress response, justifies the complexity of the virulence phenotype which was attenuated probably due to a combination of mechanisms. In this regard, the wide gene expression analysis in our study was important to globally characterize both pathogenic and non-pathogenic phenotypes and to provide the basis for future mechanistic studies. Finally, the results support our hypothesis and open a new research field for studying target genes involved in the pathogenesis, diagnosis and control of PE.

## Materials and Methods

### Cell Culture and Infection *in vitro*

The intestinal piglet epithelial cell line IPEC-J2 is a non-transformed columnar cell type derived from neonatal piglet mid-jejunum [81]. The cells were maintained in T_75_ cell culture flasks with Dulbecco’s MEM/F12 nutrient mix (1∶1) supplemented with 5% Fetal Bovine Serum, 5 ng/ml Epidermal Growth Factor (Sigma-Aldrich) and 5 ng/ml Insulin-Transferrin-Selenium mixture (BD Biosciences) without antibiotics at 37°C in a humidified atmosphere of 5% CO_2_, as previously described [15].

*L. intracellularis* isolate PHE/MN1-00 (ATCC PTA-3457) previously isolated from a pig with the hemorrhagic form of PE was used for passage 6 (pathogenic variant) and 56 (non-pathogenic variant) in cell culture. The pathogenic and non-pathogenic properties of these two variants were confirmed by experimental inoculation in pigs, performed in a previous study [59]. Pure culture of the bacteria was thawed and grown in IPEC-J2 for three continuous passages in order to allow the bacteria to recover from frozen storage. Cell culture flasks T_75_ containing 30% confluent IPEC-J2 cell monolayer were infected with 10^3^ pathogenic (passage 10) and non-pathogenic (passage 60) *L. intracellularis* organisms derived from the isolate (PHE/MN1-00). Inoculated cultures were placed in an anaerobic chamber, which was evacuated to 500 mmHg and refilled with hydrogen gas. Infected cultures were then incubated for seven days in a Tri-gas incubator with 83.2% nitrogen gas, 8.8% carbon dioxide, 8% oxygen gas and a temperature of 37°C [10].

The infection was monitored daily by counting the number of heavily infected cells (HIC) using immunoperoxidase staining with polyclonal antibody specific for *L. intracellularis*, as previously described [82]. A parallel infection was also monitored using 16-well tissue culture plates, as described in our previous study [45]. The inoculated doses in this parallel monitoring system were proportional to those used in the T_75_ flasks according to the number of IPEC-J2 cells previously passed on the day before the infection. The monitoring was achieved by counting the number of HIC daily in eight wells (replicates) infected with the pathogenic and eight with the non-pathogenic variant. Cells containing 30 or more intracellular bacteria were considered to be HIC [83], [84]. Additionally, quantitative PCR (qPCR) based on the copy numbers of aspartate ammonia lyase gene of *L. intracellularis* was performed daily as a second parameter of monitoring, as described elsewhere [45]. Direct counting of infected cells and qPCR were used to confirm the exponential phase of the bacterial growth and to standardize the amount of *L. intracellularis* used as starting material for RNA isolation. All procedures used in the present study were approved by the Institutional Sponsored Projects Administration of the University of Minnesota.

### RNA Isolation and Enrichment

On the fourth continuous passage, the infection was passed using two replicates from each variant (pathogenic and non-pathogenic) and a total of four infected monolayers were harvested. A negative control using non-infected IPEC-J2 cells was conducted by treating the monolayers with sterile complete media. On day five post-inoculation the supernatants were removed and the infected monolayers were washed with RNAprotect® Bacterial Reagent (Qiagen). The infected cells were scraped and passed through a 20-gauge needle five times. Total RNA were extracted using RNeasy® Mini Kit (Qiagen) with an additional step for removing residual DNA using DNase I (Qiagen). Total RNA were assessed by a NanoDrop ND-8000 Spectrophotometer and Agilent Bioanalyzer for quality and integrity. Only samples with RNA Integrity Numbers (RIN) ≥8.0 were used in subsequent mRNA purification steps.

The bacterial mRNA was enriched from the total extracted RNA (mixture of cells and bacterial RNA) by subtractive hybridization using MicrobEnrich™ and MicrobExpress™ kits (Ambion Inc). Briefly, oligonucleotides specific to the mammalian RNAs (18S rRNA, 28S rRNA and polyadenylated mRNAs) and to the bacterial ribosomal RNA (16S and 23S) were hybridized and captured by magnetic beads. Equal amount of input RNA was used following the manufacturer’s instructions for both pathogenic and non-pathogenic *L. intracellularis* variants. Internal controls provided in both kits were performed in all enrichments. The effectiveness of rRNA depletions was evaluated using an Agilent Bioanalyzer 2100 (mRNA Nano Series Assay).

### Library Preparation and Sequencing

The library preparation and sequencing were conducted in the core facility of the Biomedical Genomics Center at the University of Minnesota. Briefly, 100 ng of the enriched mRNAs from both pathogenic and non-pathogenic *L. intracellularis* variants were fragmented and the first and second strand sDNAs were synthetized and ends repaired. The cDNA template was enriched by PCR and validated using High Sensitivity Chip on the Agilent2100 Bioanalyzer. Following quantification of the cDNA generated for the library using PicoGreen Assay, the samples were clustered and loaded on the Illumina® Genome Analyzer GA IIx platform which generated on average 6,190,522 single reads with 76 bp. Base calling and quality filtering were performed following the manufacturer’s instructions (Illumina® GA Pipeline).

### Analysis and Mapping the Sequence Reads

Data analysis including quality control, trimming and mapping were performed in the Galaxy platform [85]. Initially, FastQC tool was applied on the raw sequence data followed by FastQ Trimmer [86]. Using these tools, ten base pairs were trimmed from the 5¢end and six from the 3¢end. As a result, trimmed sequences containing 60 bp were used in the gene expression analysis. The sequence reads showed more than 25 phred quality score [87] and were then mapped onto the *L. intracellularis* isolate PHE/MN1-00 reference genome obtained from the NCBI database using Bowtie short read aligner [88], with no more than two mismatches.

The number of reads that were mapped within each annotated coding sequence (CDS) was calculated in order to estimate the level of transcription for each gene. The Cufflinks tool was used to estimate the relative abundances of the transcript reads in each gene [89]. For comparison of the expression levels between the pathogenic and non-pathogenic *L. intracellularis* variants, the read counts were normalized based on the number of reads per kilobase of coding sequence per million mapped reads (RPKM).

### Differential Expression Analysis

Following the quantitative analysis of expressed genes, the differential expression between pathogenic and non-pathogenic variants was assessed using the CuffDiff tool [89]. The total read count was determined for each gene by combining data from the two replicate sequencing runs. RPKM values were expressed in log_2_ (RPKM) to allow for the statistical comparison of transcription levels. As a result, log_2_-fold change in abundance of each transcript was obtained by log_2_ (RPKM _[pathogenic]/_RPKM _[non-pathogenic]_). *P*-values were calculated and adjusted for multiple comparisons using false discovery rate (FDR) [90]. Significant differential expression was determined in genes with FDR-adjusted *p*-values <0.05 and fold change ±2 in the comparison of transcription levels between pathogenic and non-pathogenic variants.

### Quantitative Reverse Transcriptase PCR

The validation of the expression data identified by RNA-seq was performed by qRT-PCR from a specific set of genes: LI0005 (superoxide dismutase); LI0035 (fur regulator); LI0447 (hypothetical protein); LI0614 (thioredoxin); LI0902 (outer membrane protein); LIA017 (Fe-S oxidoreductase); LIC056 (hypothetical protein); LIB024 (chromosome partitioning ATPase); LI0825 (lipid A core-O-antigen ligase) and LI0959 (30S ribosomal protein S10). Specific primers were designed using Roche Universal Probe Library (UPL) generating a UPL probe number (Table S2). RNA samples were synthesized to first-strand cDNA using SuperScript® II RT (Invitrogen, Carlsbad, CA). Duplicate qRT-PCR reactions from each primer probe set were validated by five serial dilutions of cDNA on the ABI7900HT instrument (Applied Biosystems, Foster City, CA). After validation, quantitative PCR was performed in duplicates using 15 ng of cDNA per sample with the following conditions: 60°C for 2 min, 95°C for 5 min and 45 cycles (95°C/10 sec and 60° for 1 min). Averages of relative transcriptional levels were calculated and log_2_ transformed in order to be compared to the RNA-seq expression levels. Linear regression model was used to evaluate the correlation between average RPKM and qRT-PCR data.

## Supporting Information

Figure S1 **Reproducibility of biological replicates.** RPKM of replicate 1 plotted on the y-axis and replicate 2 on the x-axis. Each spot represents a single gene. Black circles represent genes expressed by the pathogenic isolate (PHE/MN1-00 at passage 10) and the linear regression (solid trendline −r^2^ = 0.862). Gray squares represent genes expressed by the non-pathogenic isolate (PHE/MN1-00 at passage 60) and the linear regression (dashed trend line −r^2^ = 0.813).
(TIF)

Figure S2 **RPKM representing the transcription levels (y-axis) and the number of mapped genes (x-axis) onto the** ***L. intracellularis*** **reference genome.** (A) Pathogenic variant showing 720 expressed genes. (B) Non-pathogenic variant showing 330 expressed genes. The locus tags of the four highest expressed genes are described.
(TIF)

Table S1 **Comparison between the transcription levels of genes commonly expressed by the pathogenic and non-pathogenic homologous** ***L. intracellularis*** **isolate PHE/MN1-00.**
(XLS)

Table S2 **Primers used for validation of RNA-seq expression data by quantitative reverse transcriptase PCR assay.**
(XLS)
