# Supplementary figures and images for: Comparative Transcriptional Analysis of Homologous Pathogenic and Non-Pathogenic Lawsonia intracellularis Isolates in Infected Porcine Cells
Source: PLoS One. 2012 Oct 3;7(10):e46708. doi: 10.1371/journal.pone.0046708 (PMC3463550; doi:10.1371/journal.pone.0046708)

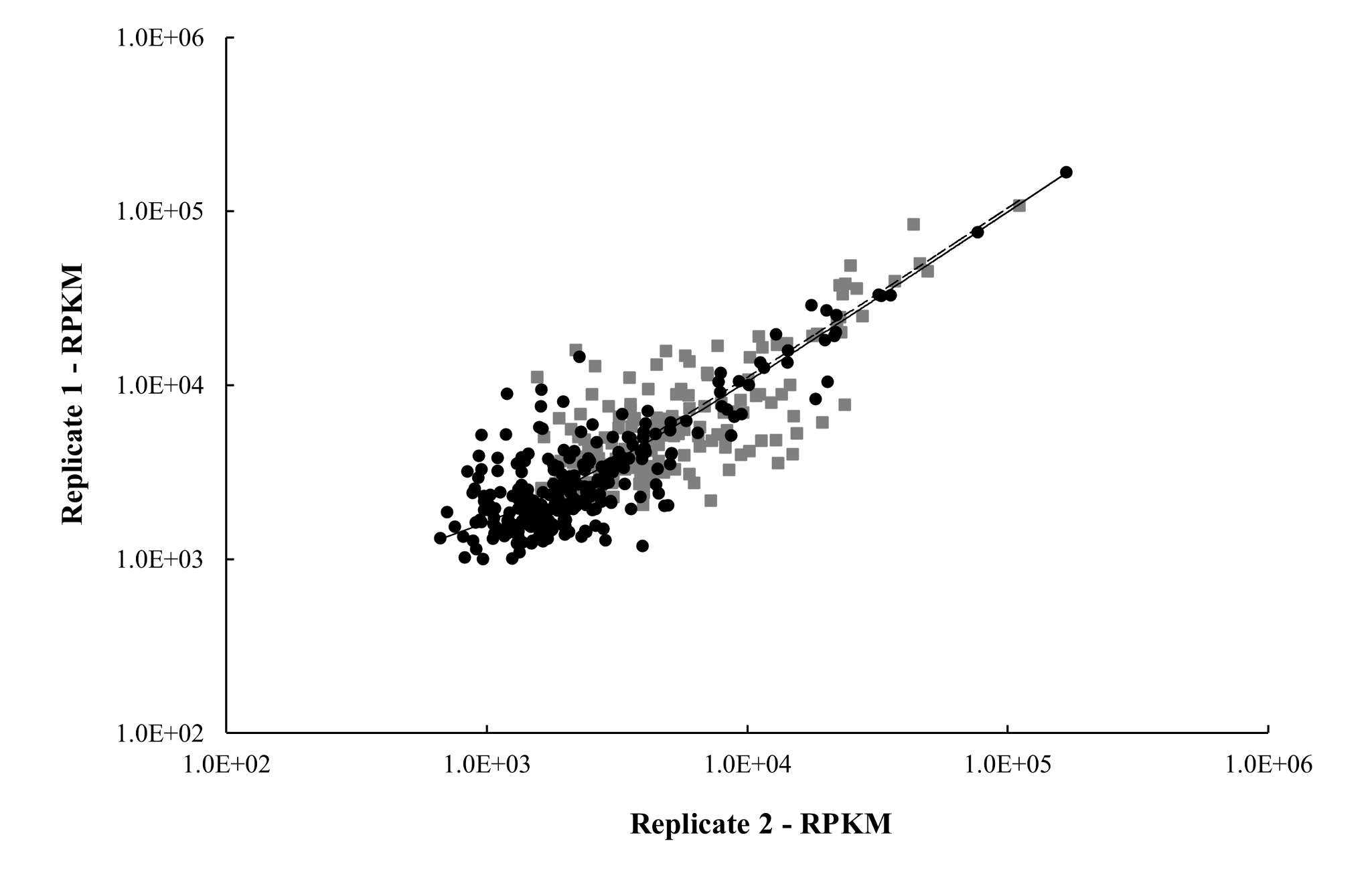

Supplement: Figure S1 — Reproducibility of biological replicates. RPKM of replicate 1 plotted on the y-axis and replicate 2 on the x-axis. Each spot represents a single gene. Black circles represent genes expressed by the pathogenic isolate (PHE/MN1-00 at passage 10) and the linear regression (solid trendline −r2 = 0.862). Gray squares represent genes expressed by the non-pathogenic isolate (PHE/MN1-00 at passage 60) and the linear regression (dashed trend line −r2 = 0.813). (TIF) [file pone.0046708.s001.tif]

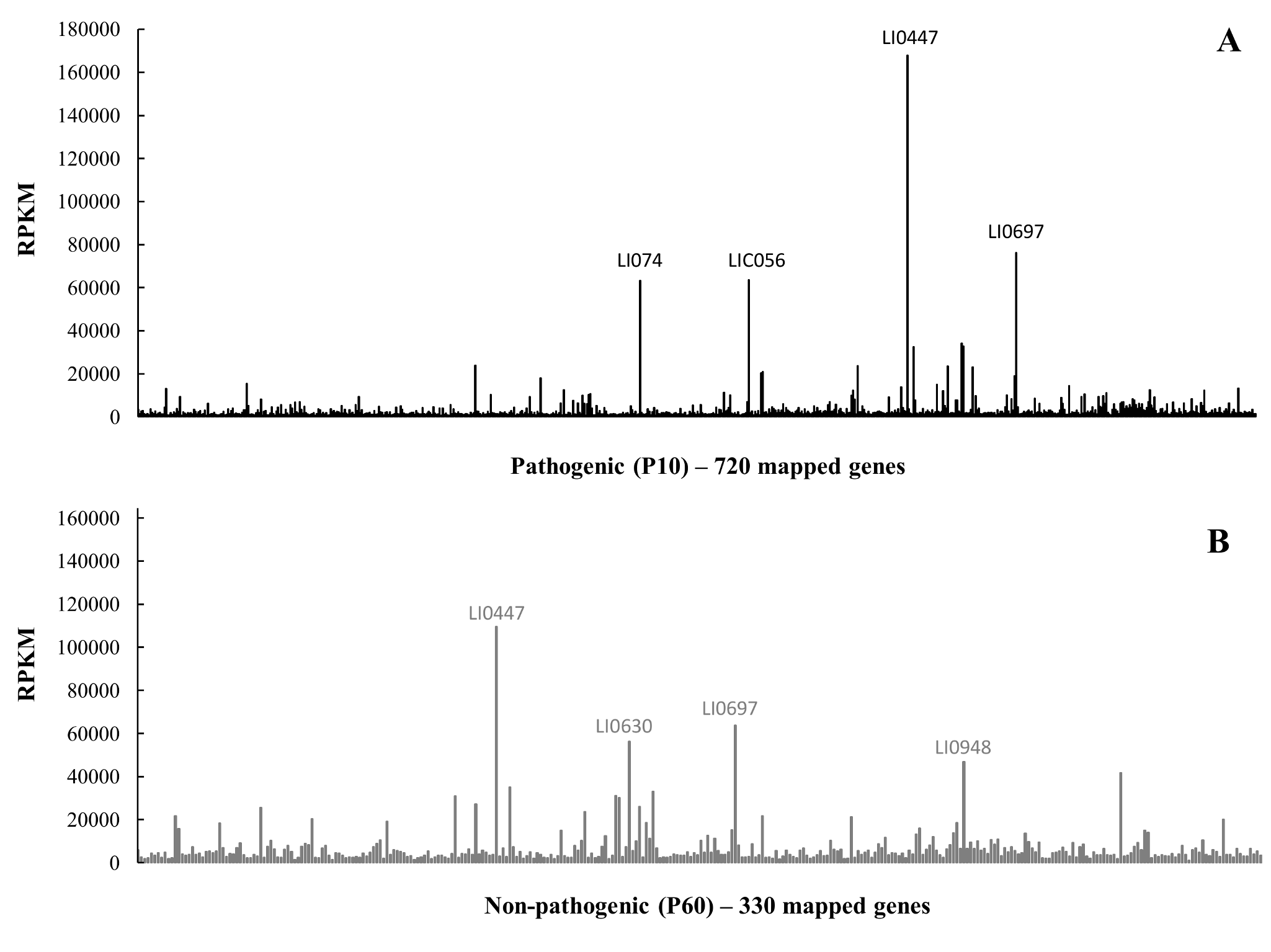

Supplement: Figure S2 — RPKM representing the transcription levels (y-axis) and the number of mapped genes (x-axis) onto the L. intracellularis reference genome. (A) Pathogenic variant showing 720 expressed genes. (B) Non-pathogenic variant showing 330 expressed genes. The locus tags of the four highest expressed genes are described. (TIF) [file pone.0046708.s002.tif]
